# Interindividual variations in associative visual learning: Exploration, description, and partition of response characteristics

**DOI:** 10.3758/s13428-023-02208-z

**Published:** 2023-08-24

**Authors:** Catherine Brandner, Elsa Raynal, Paolo Ruggeri

**Affiliations:** Brain Electrophysiology Attention Movement Laboratory, Institute of Psychology, University of Lausanne, Geopolis Quartier Mouline, CH-1015 Lausanne, Switzerland

**Keywords:** Interindividual differences, Associative learning, Signal detection theory, K-means, Principal component analysis

## Abstract

Relying on existing literature to identify suitable techniques for characterizing individual differences presents practical and methodological challenges. These challenges include the frequent absence of detailed descriptions of raw data, which hinders the assessment of analysis appropriateness, as well as the exclusion of data points deemed outliers, or the reliance on comparing only extreme groups by categorizing continuous variables into upper and lower quartiles. Despite the availability of algorithmic modeling in standard statistical software, investigations into individual differences predominantly focus on factor analysis and parametric tests. To address these limitations, this application-oriented study proposes a comprehensive approach that leverages behavioral responses through the use of signal detection theory and clustering techniques. Unlike conventional methods, signal detection theory considers both sensitivity and bias, offering insights into the intricate interplay between perceptual ability and decision-making processes. On the other hand, clustering techniques enable the identification and classification of distinct patterns within the dataset, allowing for the detection of singular behaviors that form the foundation of individual differences. In a broader framework, these combined approaches prove particularly advantageous when analyzing large and heterogeneous datasets provided by data archive platforms. By applying these techniques more widely, our understanding of the cognitive and behavioral processes underlying learning can be expedited and enhanced.

**Supplementary information:**

The online version contains supplementary material available at 10.3758/s13428-023-02208-z.

Individual differences are the main source of variability that are most often overlooked by data averaging. Interest in these variations is growing, as they may improve our understanding of the brain processes underlying cognitive functions, although knowing what type of variation affects performance remains a challenge (e.g., Kanai & Rees, 2011). Measuring interindividual variations in specific cognitive abilities and choosing an appropriate technique to group them according to their characteristics could offer a solution. To achieve this goal, we developed a shape-color association task, adapted to electrocortical recording, to track the learning process of participants without any prior instruction on how to give a correct binary response (yes or no). The response choice was based on the following: (a) that encoding and retention of visual information plays an important role in the performance of common everyday tasks and (b) that this ability is known to vary across individuals and appears to be strongly correlated with overall cognitive ability (Luck & Vogel, 2013). After all the participants completed the task, both practical and methodological issues arose. They primarily concerned the partitioning of participants into groups, based on the variations observed in their responses, to find helpful structures or patterns for characterizing the resulting groups. Accordingly, using a clearly application-oriented approach, we aimed in this article to explore the suitability of existing techniques to achieve this goal.

A considerable amount of research is devoted to individual differences. Surprisingly, however, it is not easy to find studies describing techniques or procedures for partitioning data according to their characteristics. After selecting papers dedicated to learning or memory, we were faced with a variety of methods used to assess individual differences. Concerning the correlational approach, we mainly found studies that used (a) a wide range of learning tasks and factor analysis techniques to assess associations between tasks that were used to predict individual differences in a specific task (e.g., Kane et al. 2016; Robison & Unsworth, 2017), (b) generalized linear mixed models to examine the influence of a particular independent variable on composite variables created by combining a range of learning task scores (e.g., Meier et al. 2018), or (c) structural equation modeling to assess the relationships between individual differences in working memory capacity and other cognitive abilities, or combined structural equation modeling with other modeling techniques to categorize individual differences in learning (e.g., Lewandowsky, 2011; Musso et al., 2019). Although it is difficult to find studies devoted to methods, it is relatively easy to find studies warning of the shortcomings of the use of these methods to assess individual differences. One criticism is that the tasks used to extract factors are not closely correlated with each other. Another is that these types of design submit the participants to numerous tasks that can potentially induce fatigue and decrease attention. To increase the robustness of the results it would be preferable to measure all the characteristics the behavioral response attached to a single task instead to measure response to several tasks (see, for instance, Carroll, 1978; Rouder & Haaf, 2019; Watkins, 2018).

Classic experimental approaches to individual differences in learning are usually less sophisticated. One of the most commonly encountered techniques for studying individual differences is to use the individual mean on an extra continuous predictor variable, divide it into quartiles, and finally select the upper and lower quartiles to create extreme groups, or to divide the median distribution of a continuous predictor variable and create two groups (e.g., Bleckley et al., 2003; Colflesh & Conway, 2007; Kyndt et al., 2012; Long & Prat, 2002; Lusk et al. 2009; Unsworth et al., 2004; Watson et al., 2005; Ye et al., 2021). Although this technique is simple to apply, it causes various problems. One of the most important is the conversion of a numeric variable into a categorical variable for the creation of groups, which leads to a loss of information that can distort results, as demonstrated in several works (e.g., Farewell et al., 2004; Fernandes et al., 2019; Knüppel & Hermsen, 2010; MacCallum et al., 2002; Maxwell & Delaney, 1993; Royston et al., 2006).

Other concerns have arisen in studies dedicated to individual differences the lack of detailed descriptions of the raw data before proceeding to grouping, the relevance of comparing only extreme groups by using the lower-upper quartiles, the nearly systematic exclusion of data considered as outliers, and the almost exclusive reliance on central tendencies of response time or correct response. To our view, these practices, rarely explained or justified, preclude the possibility of fully exploring individual differences.

To circumvent the limitation of behavioral responses to the mean of correct response, we used signal detection theory (SDT). This technique is ideally suited to a behavioral task designed to provide binary responses (e.g., “yes” and “no” or “correct” and “incorrect”), which is frequently the case in experimental behavioral studies. Using a four-way contingency table, SDT provides access to different response characteristics, including the rate of correct and incorrect responses, the ability to discriminate targets (signal) from lures (noise), and the decision criterion used to trigger a response (Green & Swets, 1966; Swets, 1996). To find a suitable technique for partitioning the response characteristics data set into groups, we turned to techniques from computer sciences, in particular machine learning (for an overview, see, e.g., Alpaydin, 2014, but see also Putatunda, 2019, for a short review) and data mining techniques (for an overview, see, e.g., Han et al., 2012). Clustering is a valuable exploratory technique based on an algorithm that works without categories or prior information. It provides a simple schematic representation of a data set from different variables by partitioning it into groups. The resulting clusters (groups) are created based on the principle of maximizing intraclass similarity and minimizing interclass similarity, so that data within one cluster are highly similar to each other and highly dissimilar to data in other clusters. K-means clustering (MacQueen, 1967; for a review, see Steinley, 2006) is one of the most popular clustering techniques. Based on distance measures, it is implemented in most standard statistical software. This technique allows one to find a structure or pattern from the input data set and to create qualitatively different groups. It is therefore particularly useful for studying individual differences (e.g., Hofmans, & Mullet, 2013).

Prior to proceeding with data partitioning into groups, we examined individual variability throughout the learning process by using detailed descriptive analyses of the raw data set. These analyses included classic approaches and SDT analysis. Participants were then clustered on the basis of their standardized responses by using hierarchical and k-means clustering (Clustering A). Following this first analysis, a data reduction technique, principal component analysis (PCA), was applied to the response characteristics set to extract factor scores (FS) used prior to proceeding with a second k-means clustering (Clustering B). Finally, a receiver operating characteristic (ROC) analysis, followed by k-means clustering, was used to explore the groups’ trade-off between true positive response (TPR) and false positive response (FPR) rates, as well as to provide information about the groups’ overall accuracy and the decision criterion used to trigger a response (Clustering C).

Analyses were completed by assessing the consistency of the assignment of participants to each of the three clusterings by using three standard partition comparisons, namely, Rand (Hubert & Arabie, 1985; Rand, 1971) and Jaccard (Jaccard, 1908) similarity indices and the variation of information dissimilarity index (Meilă, 2003).

## Method

### Participants

Forty-nine volunteer participants (11 men) aged 20.53 years ± 1.58 years (mean ± *SD*) provided written informed consent and participated in exchange for course credits. Participants were equivalent in terms of age, education (year of university propaedeutics), and health status (no history of neurological or psychiatric disorders or medication use, normal or corrected-to-normal vision, normal color vision). The study was approved by the Ethics Committee of the Canton of Vaud (Switzerland; protocol no. 2019-02352) and was conducted according to the Declaration of Helsinki.

### Visual stimuli and task

**Fig. 1:**
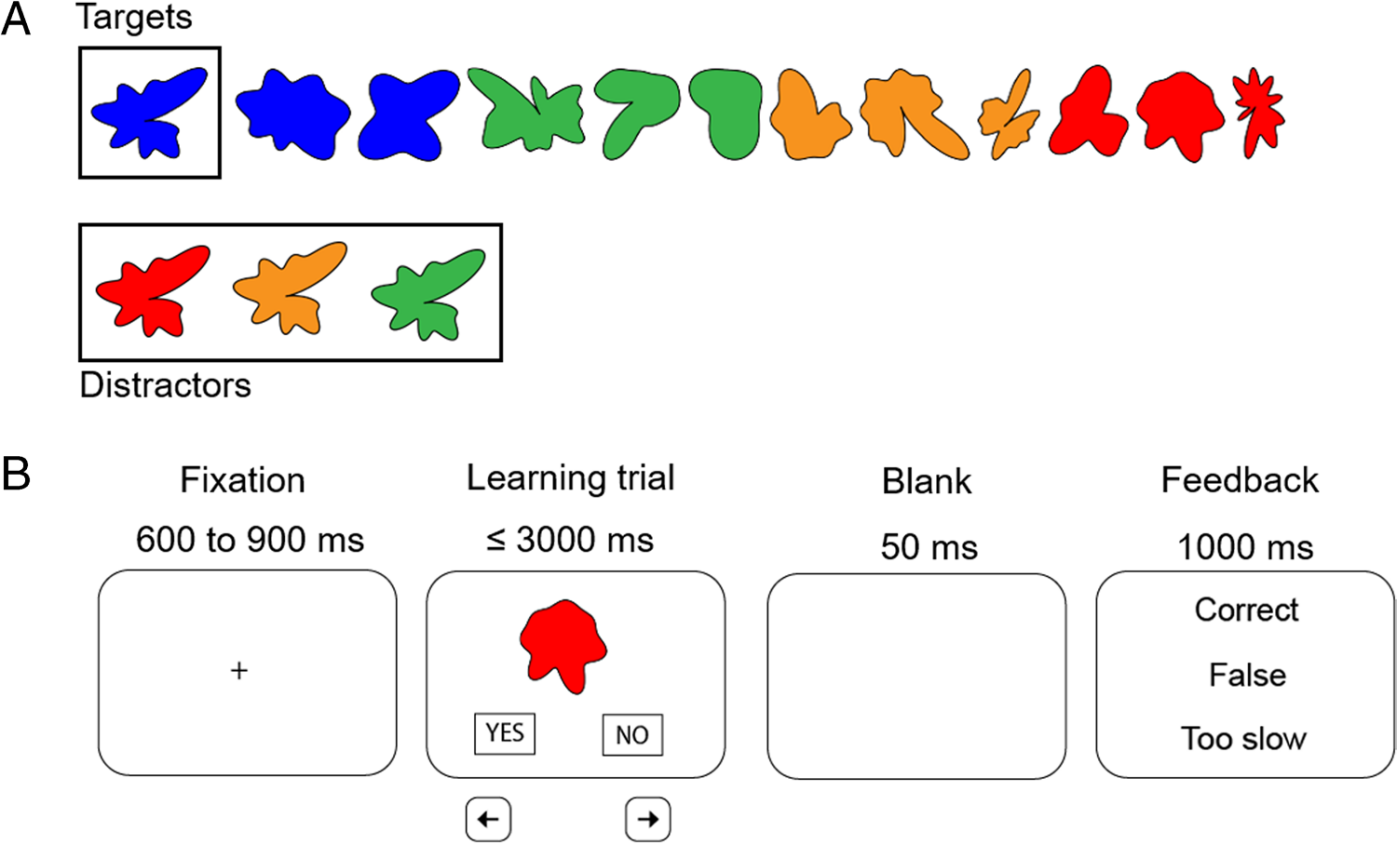
Experimental task design. **A** The 12 target stimuli created for the task, randomly assigned to each of the four colors (three blue, three green, three orange, three red), and distractors for the first stimulus (the target is associated with blue; distractors are associated with red, orange, and green). **B** Experimental scheme. The numbers represent the timing of presentation. Feedback represents the three possible response evaluations (“Correct” was displayed after a correct response, “False” was displayed after an incorrect response, and “Too slow” was displayed when no response was selected within 3000 ms)

On the basis of pretests, 12 abstract and irregular egg-shaped stimuli created with MATLAB (Version 9.3.0 R2017b) were selected to exclude stimuli too similar in form or strikingly similar to non-abstract objects (Fig. 1A). To design targets, we randomly assigned each stimulus to one of four colors (blue, red, orange, or green) and used the stimuli in remaining colors as distractors. Stimuli were presented by using PsychoPy v3.1.2 (Peirce et al., 2019) in random order to the center of 23-inch monitors at an 11° visual angle, in combination with the yes/no response choice (Fig. 1B).

### Experimental procedure

Participants were comfortably seated in a sound-attenuating booth in front of the monitor positioned at eye level. Before beginning the task, participants performed 21 test trials with stimuli that were not included in the task. During the recording session, each trial began with a fixation cross with a random duration of between 600 and 900 ms, followed by a stimulus (color-shape association) and response options (“YES” or “NO”) for 3000 ms or until a response was given. Participants endorsed or rejected the presented color-shape association by pressing the left (“YES”) or right (“NO”) arrow on the keyboard. Following a blank screen of 50 ms duration, a 1000 ms feedback screen informed the participant about whether his response was correct, incorrect, or too slow. Participants performed five blocks of 72 trials (total 360 trials). Each stimulus was distributed to occur six times in each block and was in the correct color in 50% of the trials. For each participant, we recorded the correct responses to the 360 trials.

## Descriptive analyses of the associative learning task

As indicated in the introduction, the absence of a detailed data exploration before creating groups seems an unfortunate practice. Detailed raw data descriptions provide great insights into patterns and changes that can be observed with learning and provide a better overview of individual differences. For this reason, the 360 trials were first analyzed by using 10-trial window moving averages to visualize ranked individual performance, identify good and erratic learners, and target stimuli learning trajectories. Individual averages (grand mean) of correct responses were then subjected to SDT analysis to extract eight response characteristics involved in triggering a response.

### Raw data exploration

**Fig. 2:**
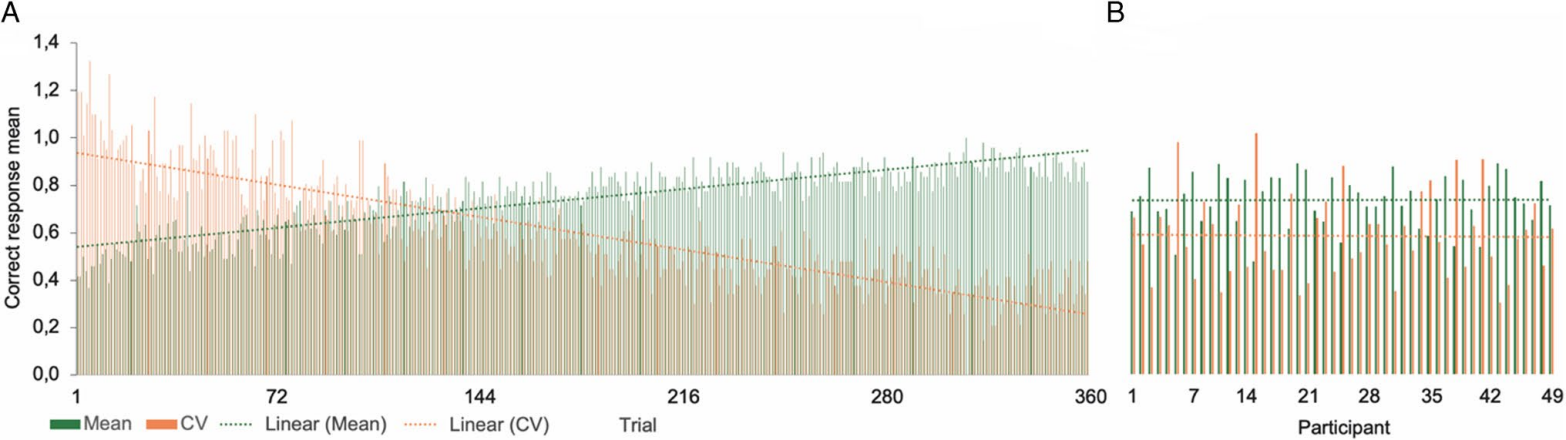
Raw correct response*.* **A** Evolution of the average correct response mean and coefficient of variation (CV) along the 360 learning trials. **B** Grand mean and CV (i.e., *SD*/*M*) for each of the 49 participants

Learning was first explored by standard correct response mean (*M*) and the coefficients of variation (CVs; i.e., *SD*/*M*) of each of the 360 trials (Fig. 2A). Means ranged from 0.39 to 1, *SDs* from 0.0 to 0.51, and CVs from 0.0 to 1.27 throughout the 360 learning trials. Results showed a slow increase in correct responses along learning trials, but a more marked decrease in CVs (Fig. 2A). Examination of the correct response grand mean of each of the 49 participants showed that it varied between 0.48 and 0.89, *SD* from 0.28 to 0.5, and CV from 0.31 to 1.02 (Fig. 2B). About 37% of the participants reached a mean of between 0.80 and 0.89 correct responses, 33% between 0.70 and 0.78, and 31% between 0.48 and 0.69.

A repeated-measures analysis of variance (ANOVA) on five 72-trial blocks confirmed a significant increase in correct response means, *F*[4,45] = 71.15, *p* ≤ .001, Wilk's Λ = 0.14, partial η^2^ = 0.86, and a significant decrease in CV, *F*[4,45] = 63.39, *p* ≤ .001, Wilk's Λ = 0.15, partial η^2^ = 0.85, throughout learning blocks.

**Fig. 3:**
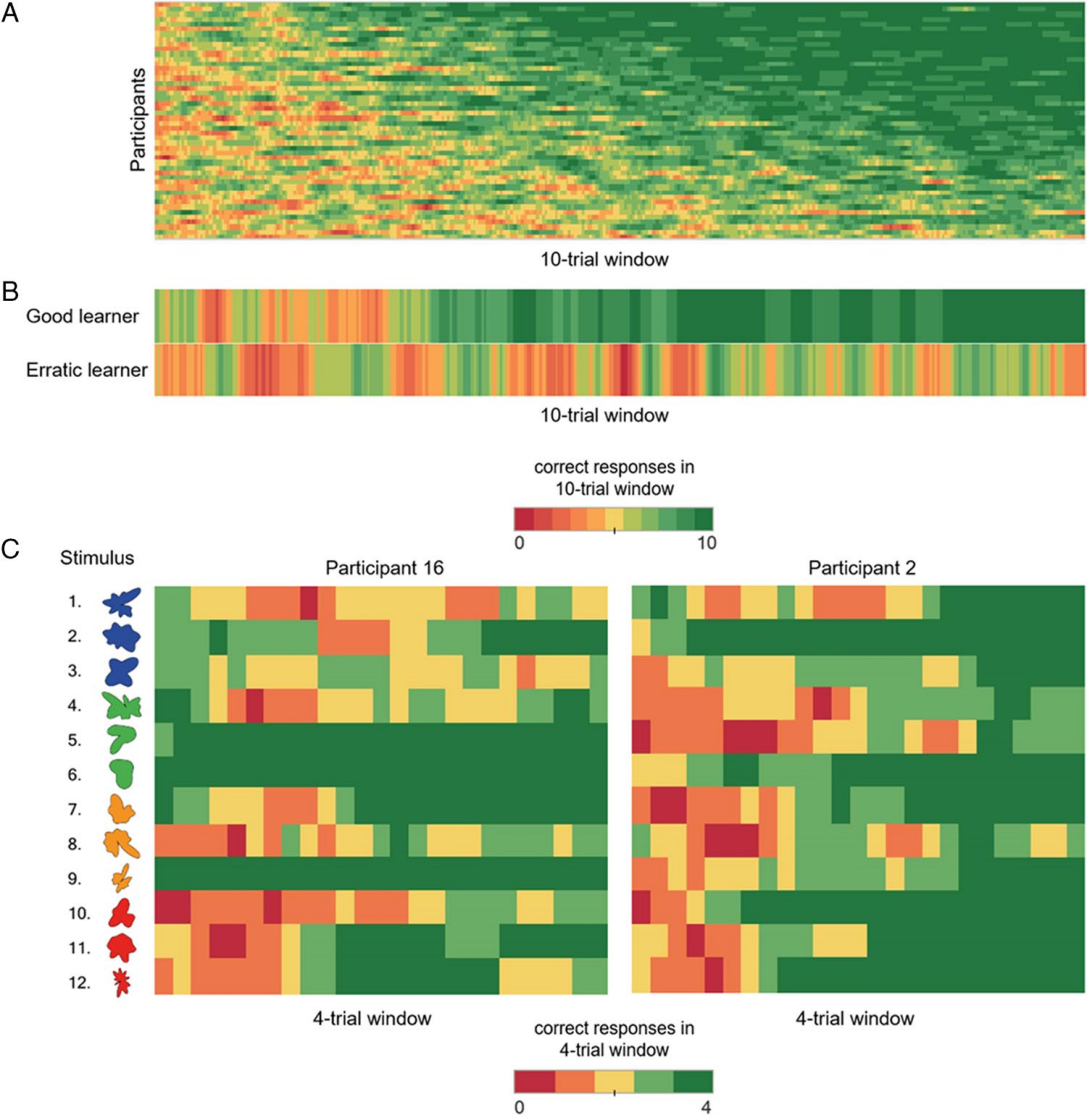
Ranked individual performances throughout learning*.* **A** Ten-trial window moving averages of correct responses for each of the 49 participants. **B** Examples of representative good (top) and erratic (bottom) learners. **C** Examples of target stimuli learning trajectory per four-trial window moving average of correct responses for each of the 12 target stimuli

This first exploration indicated that participants learned the task but at different levels. Moving averages were then used to further examine this interindividual variability. Successive 10-trial window moving means on the 360 trials for each of the 49 participants were calculated and then ranked by performance (Fig. 3A). This second exploration emphasized the large variability between participants, but also revealed learning patterns ranging from a gradual increase in correct responses, as usually observed in good learners, to erratic responses suggesting learning difficulties. As an example, two representative participants from our sample were chosen to illustrate these two learning modes (Fig. 3B). The behavior of a good learner was characterized by a higher rate of incorrect responses than correct responses at the beginning of learning, interspersed with sequences of correct responses. The learning process resulted in a progressive increase in correct responses, ending with exclusive production of correct responses. In contrast, the behavior of erratic learners was characterized by successive sequences of incorrect responses interspersed with sequences of correct responses until the end of the learning period.

To complete the exploration of raw data, we examined individual responses to the set of 12 target stimuli to verify their possible impact on the variability observed between participants. The results revealed that one of the red stimuli (Stimulus 12 in Fig. 3C) was significantly better recognized than the other stimuli (repeated-measures ANOVA, *F*(11,471) = 3.70, *p* < .001, ${\varepsilon }^{2}$ = 0.08), despite the use of pretests (see Visual stimuli and task section). Successive four-trial moving averages performed for each target stimulus over the 30 trials, where present, revealed variation between participants across the learning process, despite a comparable proportion of correct responses. Figure 3C gives an example of these variations in two representative participants with equal correct response *Ms* (*M* = 0.711 ± 0.454 and *M* = 0.716 ± 0.452). It shows that some stimuli were learned from the beginning of the learning process, whereas other stimuli were more difficult to learn.

The detailed exploration of the raw data allowed us to highlight that comparable correct response averages can obscure sizeable individual variations in the learning process. To highlight specific processes involved in the triggering of a response, we processed the raw data so that six additional response characteristic indices derived from SDT could be extracted.

### Signal detection theory

The task used in this study presented stimuli that can be either a target or a lure. The ability to accomplish such a task is based on a participant's ability to correctly discriminate targets (trigger a "yes" response in the presence of a target) and lures (trigger a "no" response in the presence of a lure).

In this framework, the use of the percentage of correct answers alone prevents the possibility of inferring the participants' detection ability. The SDT overcomes this difficulty by dissociating detection ability from the decision criterion involved in triggering a response (Green & Swets, 1966; Swets, 1996; for a review, see Wixted, 2020). A response is measured from the noise distribution or from the signal and noise distribution relative to a threshold that determines whether the stimulus is present or absent. When the proportion of the signal distribution exceeds the threshold, a “signal present” response is triggered. Conversely, when the proportion of the signal distribution is below the threshold, a “signal absent” response is triggered. Triggering a response is collectively determined by the difference between the means of signal + noise and noise alone distributions in units of the *SD*, called *d’*, and by half of the sum of signal + noise and noise alone distributions, called *c*, which depend on the threshold. Changing *d′* adjusts the distance between the distributions, whereas moving *c* adjusts the location of the threshold to release a “signal present” response. Together, these two parameters determine which signal (target) and noise (lure) stimuli will evoke a yes (present) or a no (absent) response. Relying on a low decision criterion (*c* < 0) is classically interpreted as a liberal response strategy inducing a bias toward a "yes" response, resulting in a high ability to detect true positive stimuli (target) but a low ability to detect true negative stimuli (lure). Conversely, a high decision criterion (*c* > 0) is interpreted as a conservative response strategy inducing a bias toward a "no" response, resulting in a higher ability to detect true negative stimuli (lure) but a lower ability to detect true positive stimuli (target).

In the framework of the task, each trial can result in four possible responses: release a “yes” response in the presence of a target (Hit), release a “no” response in the presence of a target (Miss), release a “yes” response in the presence of a lure (false alarm; FA), or release a “no” response in the presence of a lure (correct rejection; CR). The four stimulus-response combinations are usually summarized with a contingency table (Table 1).

**Table 1:** Confusion matrix from the four stimulus-response combinations that can be generated during the task

		Stimuli	
		Target (yes)	Lure (no)
Subject response	Yes (target)	Hit (True positive)	FA (False positive)
	No (lure)	Miss (False negative)	CR (True negative)

FA = false alarm; CR = correct rejection

All the SDT measures of performance are derived from the relationships between the signal + noise and noise distributions and can be easily calculated from the contingency table. The proportions of correct (Hit) and incorrect (FA) responses in the 360 trials of the task were calculated from the ratio of each participant’s yes response and no response. The probit transformation (inverse function of the cumulated standard normal distribution, Φ -1) was applied to the proportion of TPRs [Hit/(Hit+Miss)] and to the proportion of true negative responses (TNRs [CR/(CR+FA)] to estimate both the *d’* index (detection ability) and the *c* index (decision cutoff or criterion).

The sum of the four entries of the confusion matrix (Hit, FA, Miss, CR) of the 360 trials of the task was computed for each participant. The *d'* index was estimated by the probit transformation (inverse function of the cumulated standard normal distribution, Φ -1) of the proportion of TPRs [Hit/(Hit+Miss)]; the *c* index was estimated by the probit transformation of the proportion of TNRs [CR/(CR+FA)].

**Fig. 4:**
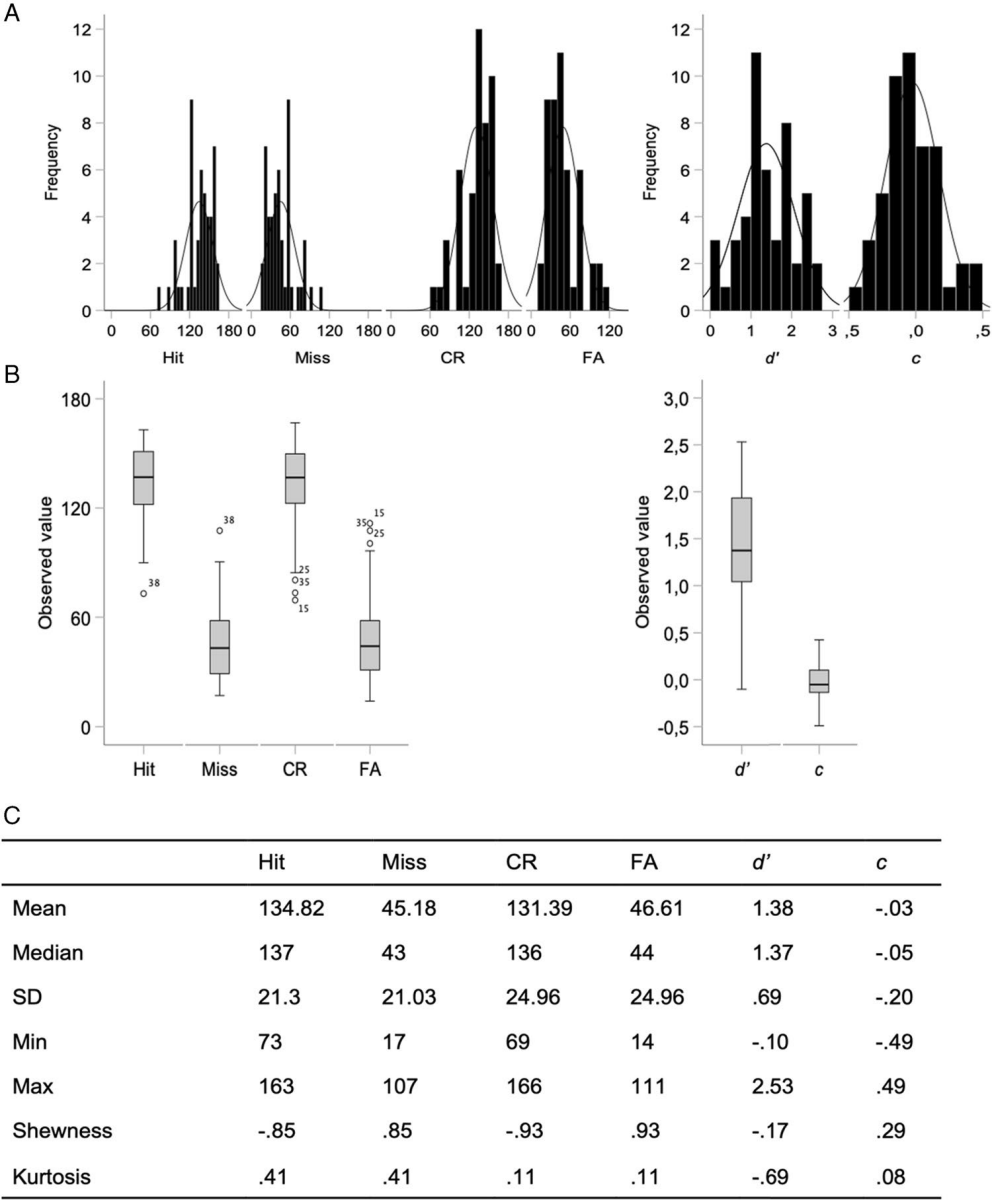
Basic statistical descriptions of the four SDT responses*.* **A** Frequency distributions of the Hit, Miss, correct rejection (CR), and false alarm (FA) response proportions (left) and of the detection ability (*d’*) and decision criterion (*c*) indices (right). **B** Boxplot descriptions of the Hit, Miss, CR, and FA with the four extreme values (outliers) compared with the mean of the responses (thick line) (left) and of the *d’* and *c* indices (right). **C** Descriptive analysis summary

Descriptive analysis of the four SDT response rates indicated that participants produced about two-thirds correct responses (TPRs and TNRs) and one-third incorrect responses (false negative responses [FNRs] and FPRs) and it revealed a large variability (Fig. 4C). This bias toward correct responses induced a leftward shift of the two Hit (skewness = −0.85) and CR (skewness = –0.93) distributions, whereas it induced a rightward shift for the two Miss (skewness = 0.85) and FA (skewness = 0.93) distributions (Fig. 4A, left, and 4C). Both *d’* (skewness = –0.17) and *c* (skewness = 0.08) distributions were symmetrical (Fig. 4B, left). The boxplots showed four values numerically distant from the rest of the sample, one under the Hit minimal values and three under the CR minimal values, which reflected the higher maximal values of Miss and FA, respectively (Fig. 4A, right), whereas *d’* and* c* were free of outlier values (Fig. 4B, center).

Here, based on the close examination of the outlier values detected by the boxplots displayed in Fig. 4B, we chose to keep them in subsequent cluster analyses considering that they were representative of natural variations that can be observed in learner groups (for details about outlier values, see, for instance, Han et al., 2012).

### Eight response characteristics

To build the response characteristics data set to be used for group partitioning, four additional indices were calculated: (1) sensitivity (Ss) corresponding to the TPR rate ([Hit/(Hit+Miss)]), (2) specificity (Sp) corresponding to the TNR rate ([CR/(CR+FA)]), (3) type I error (TI) corresponding to the FPR rate ([FA/(FA+CR)]), and (4) type II error (TII) corresponding to the FNR rate ([Miss/(Miss+Hit)]). The final data set included the correct response *M*, CV, *d’*, *c*, Ss, Sp, TI, and TII. These eight response characteristics were used for subsequent examinations.

**Fig. 5:**
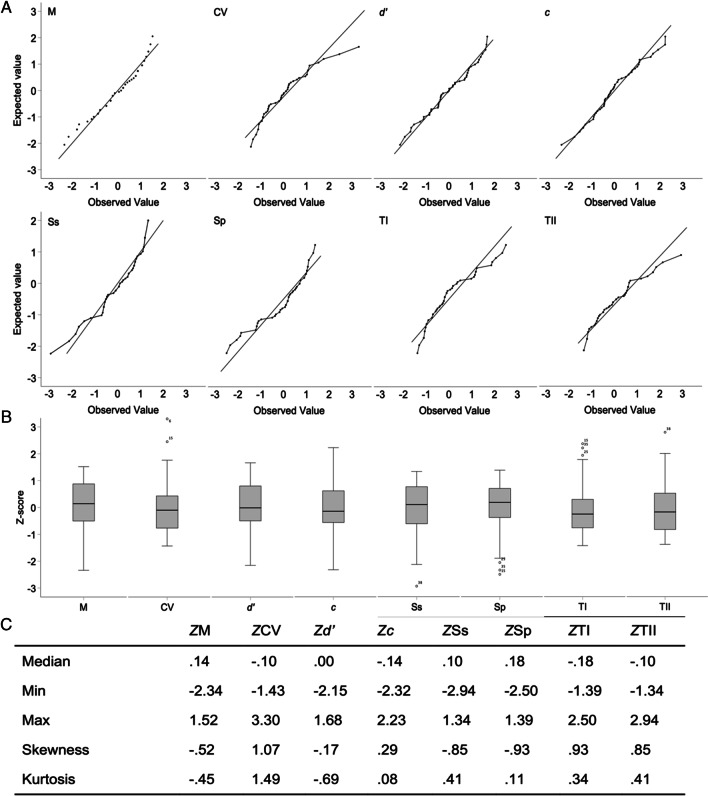
Basic statistical descriptions of the eight response characteristics*.* **A** P-P plot showing the differences between the observed percentiles (x axis) and the theoretical percentiles (y axis). **B** Descriptive analysis summary. **C** Boxplot descriptions of the eight response characteristics. M = correct response mean; CV = coefficient of variation of the correct response mean; *d*’ = detection ability; *c* = decision criterion; Ss = sensitivity; Sp = specificity; TI = type I error; TII = type II error

Descriptive analysis (Fig. 5B) of the eight learning characteristics (*z*-scores) showed few variations in the median (range from 0.0 to –0.18). P-P plots (Fig. 5A) showed that Ss (skewness = –0.85) and Sp (skewness = –0.93) were clearly left-shifted, as was *M* (skewness = –0.52) but to a lesser extent, whereas *d’* was mostly unshifted (skewness = −0.17) and CV (skewness = 1.07), TI (skewness = 0.93), and TII (skewness = 0.85) were clearly right-shifted, as was* c* (skewness = 0.29). The boxplots revealed highest minimal values in *M*, *d’*, and Ss with one outlier value, as well as in Sp with three outlier values; the highest maximal values were observed in CV with two outlier values, TI with three outlier values, and TII with one outlier value (Fig. 5C).

Preprocessing of the response characteristics data set, revealing a large variation among the participants, gave meaningful support to proceeding to data partition by using clustering analysis.

### Clustering techniques

Clustering is a technique in machine learning and data analysis that involves grouping together similar data points or objects based on their characteristics or features. This is an unsupervised learning technique (without predefined class labels or target values), using computational algorithms to discover patterns and relationships within a data set, as well as to identify distinct groups or clusters based on the similarities between their characteristics (for a review, see Jain and Dubes, 1988; Jain et al., 1999; Duda et al., 2001; Lerman, 2016; Rokach, 2010). These techniques have gained popularity through statistical software and are now used as exploratory tools to analyze multivariate data sets. Among the various clustering algorithms, we used two: (1) hierarchical bottom-up clustering with Ward's method (Ward, 1963), grouping similar data points into nested clusters based on their similarity or dissimilarity. The algorithm aims to minimize the variance within each cluster by merging the two clusters that result in the smallest increase in the total sum of squared differences within clusters. This process is repeated until all data points belong to a single cluster or a predetermined number of clusters is reached; and (2) centroid-based clustering k-means, partitioning a data set into k clusters based on their similarity. The algorithm works by randomly selecting k initial cluster centers, assigning each data point to the nearest cluster center, and then updating the cluster centers based on the mean of the data points assigned to each cluster. This process is repeated iteratively until the cluster assignments converge or a stopping criterion is met. Analyses were performed with SPSS (version 27.0.1.0).

### Hierarchical clustering

This technique creates a hierarchical decomposition of a given set of data that can be classified as being either agglomerative or divisive, depending on how the hierarchical decomposition is formed. The agglomerative approach starts with each data set forming a separate group and successively merges the data close to one another, until all groups are merged into one (the topmost level of the hierarchy). This hierarchy can be visualized by a branching diagram (dendrogram) representing the hierarchy of groups based on the degree of similarity between the data (see, for instance, Hastie et al., 2009; Maalel et al., 2014). Agglomerative hierarchical clustering (HC) was performed on the eight response characteristics in a data set by using a range of solutions (two to six clusters). To maximize within-cluster homogeneity, we used the Ward’s method and squared Euclidean distance as an interval measure. The number of clusters to use for k-means clustering was determined from examination of both the dendrogram and the agglomerative coefficient of the HC (cutoff at the change of the slope).

### K-means clustering

K-means is the most widely used centroid-based clustering algorithm for identifying and aggregating data into a set of relatively homogeneous clusters based on their similarity (e.g., Greenacre & Primicerio, 2014; MacQueen, 1967; Murtagh & Contreras, 2012; Steinley, 2006). To use this clustering procedure, the number of clusters (k) first needs to be predefined by HC analysis. Data are clustered from the shortest Euclidean distance of each data point to the k-centers. This method compares objects from each cluster (based on Euclidean distance between each case and the mean [center] of cases in each cluster) and reassigns incorrectly classified objects to a more suitable cluster by successive iterations to decrease within- and between-cluster variance.

#### K-means clustering analysis of eight response characteristics (Clustering A)

From the HC coefficient measures of similarity, a three-cluster solution was retained. The three-cluster k-means analysis was performed on the normalized (*z*-score) data set of the eight response characteristics. Convergence was achieved due to no change (.000) in cluster centers after five iterations. The minimum distance between initial centers was 6.81. Table 2 shows distances between final centers of the three clusters and points. The farthest distance was observed between Clusters 1 and 3.

**Table 2:** Final distances between cluster centers of clustering A and number of cases in each of the three clusters (N)

Cluster	1	2	3
1		3.51	4.82
2	3.51		3.55
3	4.82	3.55	
*N*	13	13	23

**Fig. 6:**
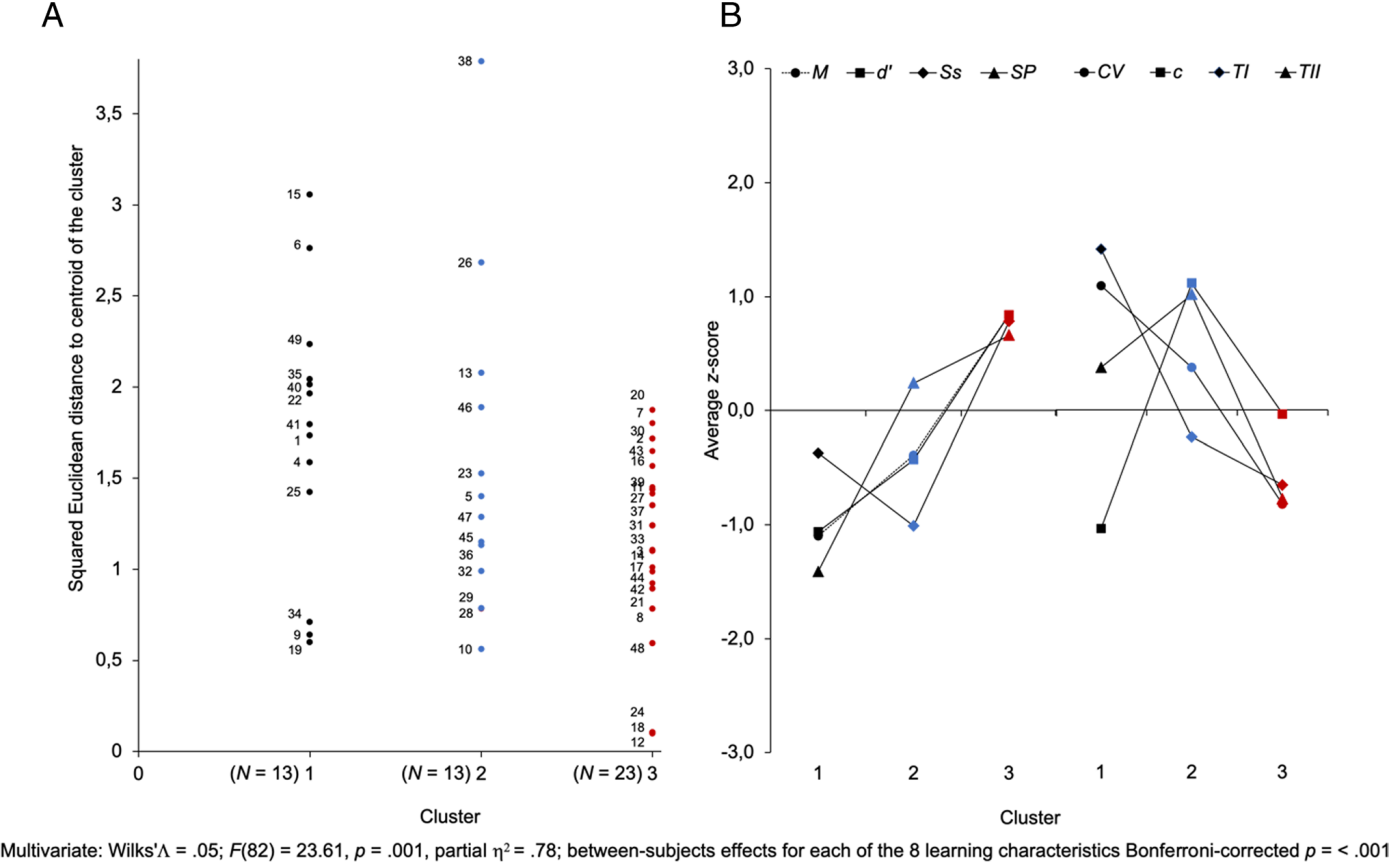
K-means clustering analysis of the eight response characteristics (Clustering A). **A** Assignment of participants to each of the three clusters. **B** Profiles of the averages of the eight response characteristics (*z*-score) of each cluster. Left: *M* = correct response mean; *d'* = detection ability; Ss = sensitivity; Sp = specificity. Right: CV = coefficient of variation of the correct response mean; *c* = decision criterion; TI = type I error response rate; TII = type II error

Squared Euclidean distances to the centroid of each of the three clusters were recorded and used to visualize the distribution of participants in each of the three clusters (Fig. 6A). The average (*z*-scores) for each response characteristic of each cluster was computed and plotted to visualize the profiles of each characteristic and estimate the global strategy used to release a response (Fig. 6B).

K-means partitioning divided the data set into two clusters, each encompassing 26% (*N* = 13, Groups 1 and 2) of the participants, and a larger cluster encompassing 47% (*N* = 23, Group 3) of the participants (Fig. 6A). The profiles of the averages of the eight characteristics (Fig. 6B) indicated that participants in Group 1 were characterized by the lowest correct response *Ms*, *d'*, and Sp of the sample, as well as a lower than mean Ss associated with the highest averages of TI and CV, a higher than mean TII, and a lower than mean *c*. Group 2 was characterized by a lower than correct response *M* and *d′*, the lowest Ss of the sample, and a slightly above mean Sp associated with a slightly above mean CV, a slightly below mean TI, and the highest *c* and TII of the sample. Group 3 was characterized by the highest correct response *Ms*, *d′*, Ss, and Sp of the sample associated with the lowest averages of CV, TI, and TII of the sample and a just below the mean *c*.

To clarify this first picture of group partitioning, we subjected the eight learning indices data set to a PCA.

## Dimensionality reduction technique

### Principal component analysis (PCA)

PCA is an exploratory statistical tool used to reduce the dimensionality of a set of interrelated variables while retaining maximal explained inertia. This technique allows one to assess the number of independent dimensions (principal components) underlying the variables, as well as visualization by projecting the extracted information into a lower-dimensional subspace (e.g., Han et al., 2012; Witten & Frank, 2005). Furthermore, it allows for summarization of the between-variable relationships into standardized FS—or component scores—indicating the relative position of each observation on the latent factors and use of them for further analyses (e.g., Gorsuch, 2015; for a review, see DiStefano et al., 2009; but see Steinley, 2006, for their use in k-means clustering).

In summary, the results of the PCA performed on the eight response characteristics confirmed strong relationships among them (Table 3).

**Table 3:** Correlation matrix of the eight response characteristics

	M	CV	*d’*	*c*	Ss	Sp	TI	TII
M	1.00	−1.00	.99	.17	.81	.88	−.88	−.81
CV	−1.00	1.00	−.99	−.16	−.82	−.88	.88	.82
*d’*	.99	−.99	1.00	.15	.82	.87	−.87	−.82
*c*	.17	−.16	.15	1.00	−.43	.61	−.61	.43
Ss	.81	−.82	.82	−.43	1.00	.44	−.44	−1.00
Sp	.88	−.88	.87	.61	.44	1.00	−1.00	−.44
TI	−.88	.88	−.87	−.61	−.44	−1.00	1.00	.44
TII	−.81	.82	−.82	.43	−1.00	−.44	.44	1.00

*M* = correct response mean; CV = coefficient of variation; *d'* = detection ability; *c* = decision criterion; Ss = sensitivity; Sp = specificity; TI = type I error; TII = type II error

The communities after varimax rotation indicated that each of the eight response characteristics explained almost all of the total variance (> 99%). More precisely, the percentage of variance explained by Dimension 1 was about 74% and that by Dimension 2 about 26%.

Considering the loadings of each original variable (Table 4), Component 1 can be interpreted as a contrast between Ss and TII, Component 2 as a contrast between Sp and TI.

**Table 4:** Loadings for each original variable in the component matrix after varimax rotation with Kaiser normalization

	Component	
	1	2
Ss	1.00	.07
TII	−1.00	−.07
*d’*	.78	.62
CV	−.78	−.63
M	.77	.63
TI	−.38	−.92
Sp	.38	.92
c	−.49	.87

*M* = correct response mean; CV = coefficient of variation; *d'* = detection ability; *c* = decision criterion; Ss = sensitivity; Sp = specificity; TI = type I error; TII = type II error

**Fig. 7:**
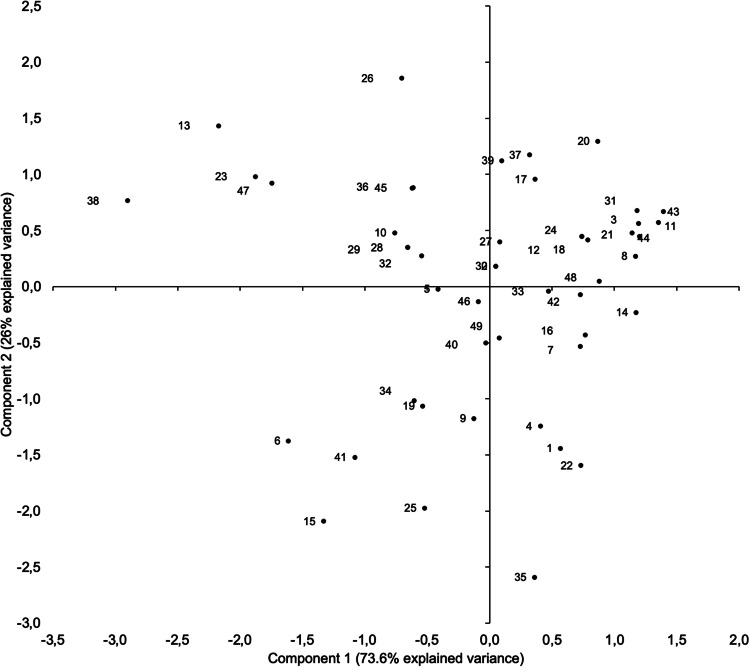
Projection of the 49 individual scores indicating the relative position of each observation in the reduced space of the first and second components of the principal component analysis

Consistent with such an interpretation, the plot of factor scores (FS) 1 and 2 (Fig. 7) clustered participants’ data points by combining TII (FS1, C1 −) with Sp (FS2, C2 +) in the upper left quadrant, Ss (FS1, C1 +) with Sp (FS2, C2 +) in the upper right quadrant, TII (FS1, C1 −) with TI (FS2, C2 −) in the lower left quadrant, and Ss (FS1, C1 +) with TI (FS2, C2 −) in the lower right quadrant.

Examination of Fig. 7 suggests that participant data points could be clustered into three groups: a group combining TII with Sp (upper left quadrant), a group combining Sp with Ss (upper right quadrant), and a group combining TII and TI (mainly lower left quadrant). This suggestion was explored by k-means clustering performed on the two FS of the PCA analysis.

### K-means clustering of FS (Clustering B)

As previously described, HC was performed on the FS data set to determine the number of clusters to use. To minimize within-cluster variance, we used the Ward method with squared Euclidean distance. From examination of the figure of the coefficient measures of similarity, a three k-means cluster analysis was performed on the data set of the two FS.

Convergence was achieved due to no change (.000) in cluster centers after three iterations. The minimum distance between initial centers was 3.62. Table 5 shows distances between final centers of the three clusters with the farthest distance between Cluster 3 and Cluster 2.

**Table 5:** Final distances between cluster centers of Clustering B and number of cases in each of the three clusters (N)

Cluster	1	2	3
1		2.09	1.91
2	2.09		2.54
3	1.91	2.54	
*N*	27	11	11

**Fig. 8:**
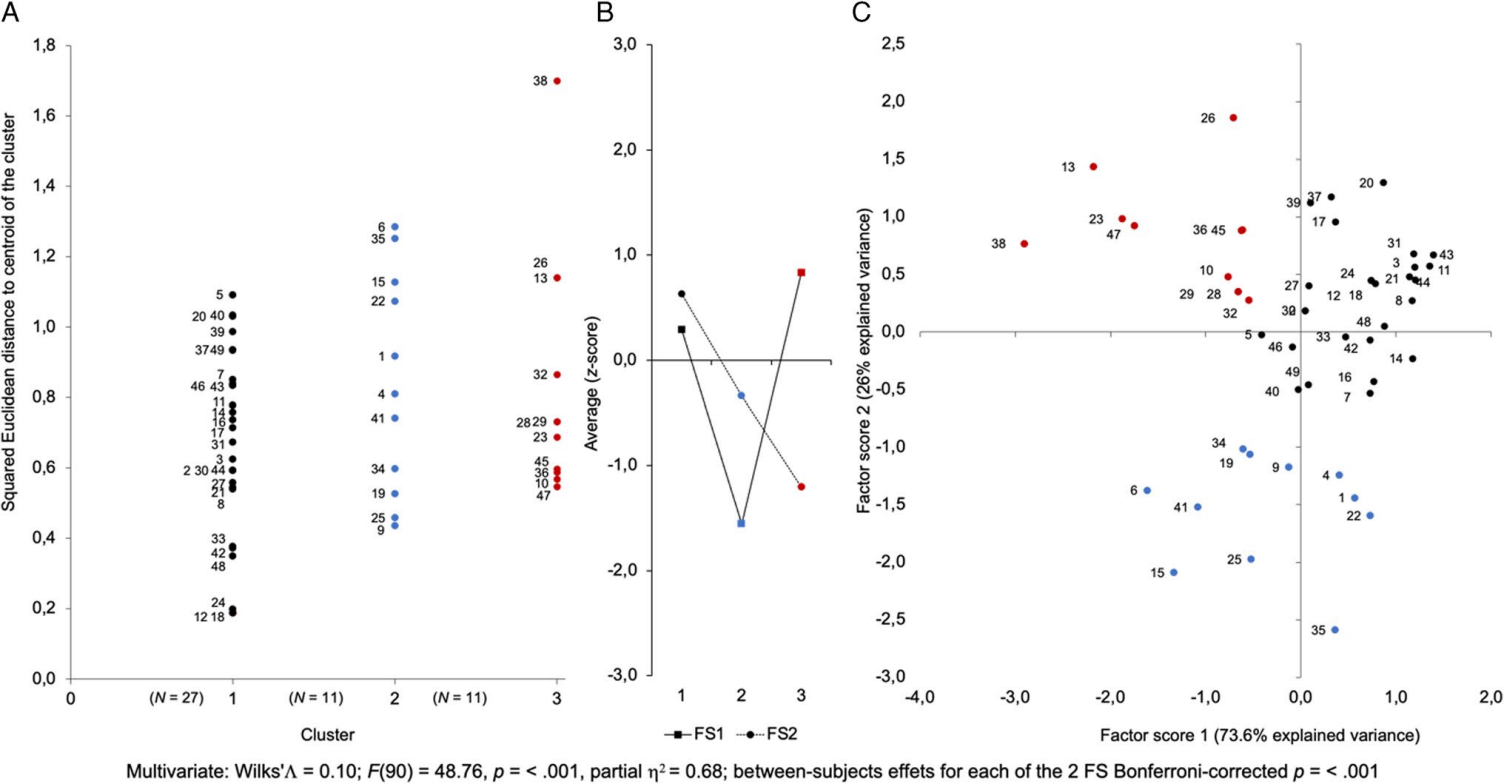
K-means clustering analysis of the two PCA factor scores (Clustering B). A Assignment of participants to each of the three clusters. **B** Profiles of the two FS averages (*z*-score) of each cluster. **C** Visualization of the assignment of each participant to their respective k-means cluster in the reduced space of the first and second components of the principal component analysis (PCA)

Squared Euclidean distances to the centroid of each of the three clusters were recorded and used to visualize the distribution of participants in each of the three clusters (Fig. 8A). The average FS (*z*-scores) of each of the three clusters was computed and plotted to visualize the groups’ profiles (Fig. 8B). Finally, the assignment of each participant to their respective k-means clusters was plotted in the factor space (Fig. 8C).

K-means partitioning divided the data set into a large cluster encompassing 55% (*N* = 27, Group 1) of the data and two clusters each encompassing 22% (*N* = 11, Groups 2 and 3) of the remaining data (Fig. 8A). The profiles of the two FS averages (*z*-scores) indicated that Group 1 was characterized by a higher than mean FS1 and FS2, Group 2 by a lower than mean FS1 and FS2, and Group 3 by a higher than mean FS1 and lower than mean FS II (Fig. 8B). The scatterplot of the individual FS allows characterization of each of the three clusters (Fig. 8C). Group 1, spreading mainly over the upper right quadrant, was characterized by high Ss and Sp; Group 2, spreading mainly over the lower left quadrant, was characterized by high TI and TII; and Group 3, spreading over the upper left quadrant, was characterized by high TII and Sp.

To complete the data exploration, we performed a final k-means clustering to assess the trade-off between managing TPR (Ss) and FPR (TI) rates.

## Receiver operating characteristic space

**Fig. 9:**
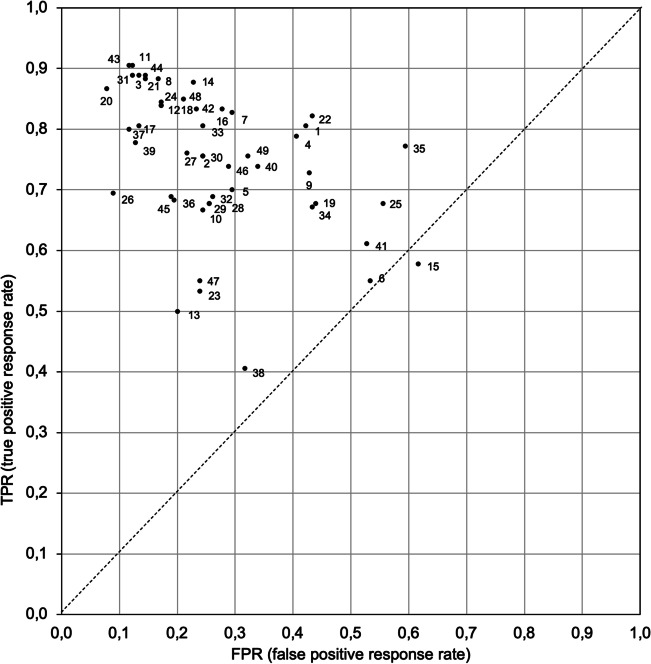
ROC space representing the response trade-off of each participant. *Note.* Each point in the ROC space represents the individual true positive response (TPR) rate: ([Hit/(Hit+Miss)], y-coordinate) plotted against the individual false positive response (FPR) rate ([FA/(FA+CR)], x-coordinate). The value of each coordinate provides the trade-off between the overall accuracy and the decision criterion used by each participant. The point at the upper left corner represents the optimal trade-off. whereas the point at the lower right corner represents the worst trade-off with only incorrect responses. The ascending diagonal (connecting 0;0 and 1;1) represents a random response. Above this diagonal, responses are better than random; below this line, responses are poorer than random. ROC = receiver operating characteristic; FA = false alarm; CR = correct rejection

The statistics of TPR (Hit) and FPR (FA) rates provide another technique for characterizing the responses of participants tested in a task that required them to learn to discriminate target and lure stimuli by giving a binary yes or no response. The individual trade-off between a TPR rate ([Hit/(Hit+Miss)]) and an FPR rate ([FA/(FA+CR)]) provides information about both the learner's overall accuracy and the decision criterion used to trigger a response (Green & Swets, 1966; Swets, 1996; but see also Fawcett, 2006; Flach, 2003). Plotting the value of each coordinate in the ROC space allows visualization of this trade-off for each participant (Fig. 9).

### K-means clustering of two-dimensional ROC space (Clustering C)

Again, the ROC space data set (TPR rate and FPR rate) was subjected to HC and k-means analyses to examine participants’ classification on these two dimensions. From examination of the figure of the coefficient measures of similarity, a three k-means cluster analysis was performed on the data set of the ROC space.

Convergence was achieved due to no change (.000) in cluster centers after two iterations. The minimum distance between initial centers was 3.63. Table 6 shows distances between final centers of the three clusters with the farthest distance between Cluster 3 and Cluster 2.

**Table 6:** Final distance between cluster centers of Clustering C and number of cases in each of the three clusters (N)

Cluster	1	2	3
1		2.41	2.18
2	2.41		2.59
3	2.18	2.59	
*N*	12	4	33

**Fig. 10:**
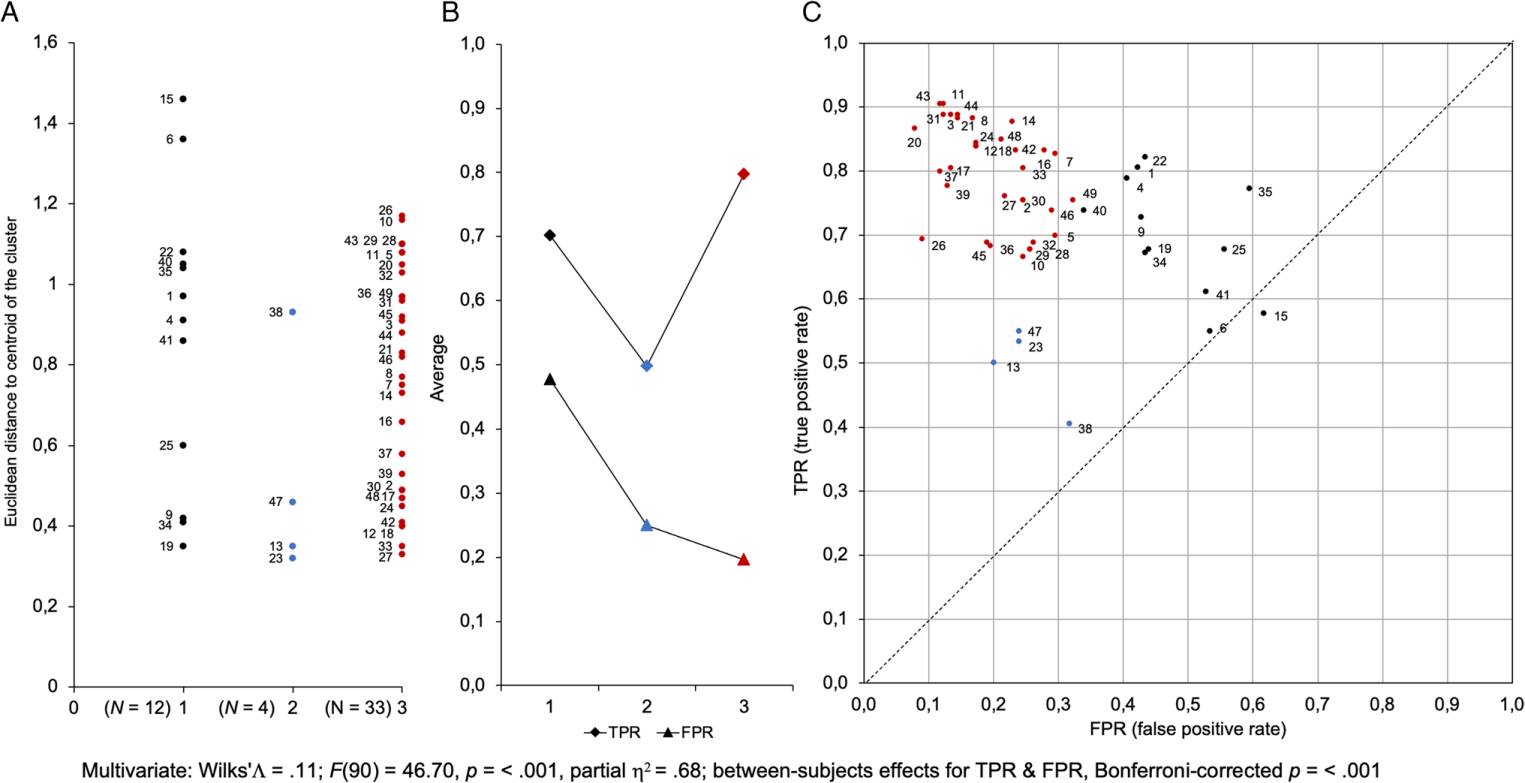
K-means clustering analysis of the true positive response rate and false positive response rate of the ROC space (Clustering C). **A** Assignment of participants to each of the three clusters. **B** Profiles of the TPR and FPR average of each cluster. **C** Visualization of the assignment of each participant in their respective k-means cluster in the receiver operating characteristic (ROC) space (see Fig. 9 for more details)

Squared Euclidean distances to the centroid of each of the three clusters were recorded and used to visualize the distribution of participants in each of the three clusters (Fig. 10A). The average rate for Ss (TPR) and TI (FPR) of each of the three clusters was computed and plotted to visualize the groups’ profiles (Fig. 10B). Finally, the assignment of each participant to their respective k-means clusters was plotted in the ROC space to estimate groups' overall accuracy and the decision criterion used to trigger a response (Fig. 10C).

K-means partitioning divided the data set into a large cluster encompassing 67% (*N* = 33, Group 3) of the data, a medium cluster encompassing 24% (*N* = 12, Group 1) of the data, and a small cluster encompassing 8% (*N* = 4, Group 2) of the remaining data (Fig. 10A).

The profiles of TPR and FPR indicated a high TPR in Group 1 with high FPR, a low TPR with a low FPR in Group 2, and a high TPR with a low FPR in Group 3 (Fig. 10B). The scatterplot (Fig. 10C) showed that the data points spread from just above the diagonal to the upper left corner, indicating that, on average, participants managed the trade-off between TPR and FPR and performed better than random, except for one who performed less well than random (Participant 15).

Confusion matrices from the averages of the four stimulus-response combinations were then used to compute accuracy and kappa (k) coefficient (Cohen, 1960) of each of the three groups. Accuracy measures the ratio of correct responses (here Hit + Correct rejection) to the total number of giving responses (here Hit + False Alarm + Miss + correct rejection). This measure, however, does not consider the possibility of randomly giving correct responses, which can be an issue when the classes are imbalanced as learning does. In such a case, the kappa coefficient – which varies from 0 to 1 – makes a more reliable measure since it considers the agreement between the observed and the randomly expected responses.

The accuracy of Groups 1 (61% of correct responses) and 2 (62% of correct responses) was not far above chance level (50%) whereas that of Group 3 was higher (80% of correct response). As the confusion matrices response classes were imbalanced, a k coefficient was used. When the level of chance was considered, the performance of Groups 1 and 2 fell to 22% and 25% correct responses, respectively, and that of Group 3 to 60% (Table 7). These poor performances have been, at least in part, due to the difficulty of the task.

**Table 7:** Average of the four stimulus-response combinations of the confusion matrix for each of the three groups

Group	1	2	3
Hit	126.33	90.10	143.39
FA	85.92	89.00	35.52
Miss	53.67	44.42	36.61
CR	94.08	134.50	144.48
*Accuracy*	.61	.62	.80
*k*	.22	.25	.60

Precision values range from 0 to 100% (perfect performance); k-values range from 0 to 1

As the responses given also depend on the decision criterion used to trigger a response, the average response strategy of each of the three groups was examined. Table 7 shows that Group 1 detected target stimuli better than lure stimuli, Group 2 detected lure stimuli better than target stimuli, and Group 3 detected target stimuli as well as lure stimuli. According to the classical interpretation, Group 1 relied on a so-called liberal response strategy which biases the response toward "yes" responses (c = −0.24), Group 2 on a so-called conservative response strategy which biases the response toward "no" responses (c = 0.35), whereas Group 3's responses were unbiased (c = 0.01).

To end exploration, we assessed the similarity between the three clustering approaches (A, B, and C) by using the usual comparison indices.

## Clustering comparison

Consistency between the three clusterings was assessed by three typical indices used for comparison between partitions. This was done by using the R package “partitionComparison.” The Rand similarity index (Hubert & Arabie, 1985; Rand, 1971) gives the proportion of pairs of individuals either belonging to the same groups in both partitions, or belonging to different groups in both partitions. The Jaccard similarity index (Jaccard, 1908) gives the same proportion after discarding pairs of individuals belonging to different groups in both partitions. Both indices range within [0 to 1]. By contrast, the variation of information dissimilarity index (Meilă, 2003) measures in nats (natural unit of information) the sum of the conditional entropies of a partition given the other, that is, the proper information carried by both partitions. It ranges within [0 to log (m) + log (m’)] where m and m’ count the number of clusters in the two partitions. As expected, Clusterings A and B were the most similar (Rand index: 0.88; Jaccard's coefficient: 0.72; variation of information dissimilarity index: 0.85), confirming that the reduction of the original data set by PCA analysis did not affect clustering. Clustering A and C and B and C were also similar (A,C Rand Index: 0.76; Jaccard coefficient: 0.56; variation of information dissimilarity index: 0.74; B,C Rand Index: 0.79; Jaccard coefficient: 0.62; variation of information dissimilarity index: 0.78), albeit A and C to a lesser degree than B and C. Taken as a whole, these results suggest good consistency between partitions despite the fact that the size of some groups varied considerably, such as in Clustering C.

## Synthesis and discussion

Finding an effective procedure for partitioning response characteristics from a visual association task into homogeneous groups to better describe learning interindividual variations was the objective of this paper. From the selected literature, one of the most encountered techniques is to create extreme groups from quartiles of measurements obtained from an additional task, or to split the median distribution of a continuous predictor variable to create two groups. To remedy these shortcomings, a more suitable technique was found in machine learning and data mining literature, and so we used hierarchical and k-means clustering on our data sets.

Exploration of the correct responses confirmed that the task was learned, but it also revealed large variability between participants. Further digging by using moving averages provided a grasp of individual differences that can be observed over the course of the task. In particular, it revealed differences in learning patterns ranging from a gradual increase in correct responses, as usually observed in good learners, to erratic responses, suggesting more reduced visual associative learning abilities. Basic statistical descriptions of the four SDT responses allowed visualization of response distributions and their skewness and the smallest and the largest values, as well as identification of extreme values. On average, about two-thirds of correct and one-third of incorrect responses were observed to induce right and left skewness, respectively, whereas *d’* and *c* distributions were symmetric. Analysis also revealed few outlier values in the CR rate (smallest values) and in an FPR rate (largest values). The same analysis of the eight learning characteristics revealed a steady median throughout responses. Ss, Sp, and correct response *M* distributions showed a negative skewness, whereas it was positive for CV, TI, and TII. The largest minimal values were observed in correct response M, Ss, and *d’*, whereas the largest maximal values were observed in CV, TI, and TII. These initial descriptive analyses allowed detailed data exploration and highlighted the variability of performances, while indicating that on average the task was learned. They brought a good basis to creation of homogeneous groups from response characteristics by using clustering analysis.

The three k-means clustering performed on the eight learning characteristics (Clustering A), on the two FS from the PCA (Clustering B), and on the TPR and the FPR rates only (Clustering C) resulted in three groups with significantly different means. The groups in Clustering A encompassed 47%, 26%, and 26% of the data. The largest group was characterized by the highest averages of correct response M, Ss, Sp, and *d’* associated with the lowest average CV, TI, and TII of the sample, as well as a *c* just below the mean. The other two groups were characterized by weaker performances. One group was characterized by the lowest averages of correct response M, Sp, and *d’* associated with the highest averages of TI and CV, while the other was characterized by the lowest Ss of the sample. The groups in Cluster B encompassed 55%, 22%, and 22% of the data. Proceeding to data reduction through PCA provided a clearer picture of the group characteristics. The largest group was characterized by high Ss and Sp. The other two groups were characterized by high TI and Sp and high TI and TII. The three groups in Clustering C encompassed 67%, 24%, and 8% of the data. Visualization of the data points indicated that the participants performed better than chance except for one. The largest group was characterized by the finest management of the trade-off between TPR and FPR rates in the sample. The medium group was characterized by a relatively high TPR rate coupled with the highest FPR rate of the sample. The small group was characterized by the lowest TPR and FPR of the sample. Although the size of the groups in each of the three clusterings varied, the results of the Rand, Jaccard, and variation of information indices supported the similarity between these three clusterings.

Overall, these results demonstrated that the SDT is a valid tool to explore the response characteristics in depth that are recorded in tasks requiring binary responses. The features gathered from SDT analysis proved to be suitable for exploring individual differences by using the k-means clustering procedure. This technique helped to characterize individual differences from the patterns observed in each of the three resulting groups. These patterns have shed light on their general attributes, which can be summarized as follows. The largest group was characterized by the highest ability to correctly learn both stimulus types (target and lure). This capacity was also characterized by a good ability to manage the trade-off between TPRs and FPRs. A second group was characterized by a good ability to correctly learn lure stimuli associated with difficulty in correctly identifying target stimuli. Regarding error type, this difficulty appeared to rely on a higher decision criterion, inducing an increase of a “no” response in the presence of a stimulus. The last group was mainly characterized by the two types of error, which could indicate that the task was not yet fully acquired.

As a reminder, the techniques briefly reviewed in the introduction highlighted correlational and experimental approaches as the primary means of assessing individual differences. Correlational techniques focus on the relationships between measures and categorical variables in questionnaires or test batteries to predict interindividual variation for a specific cognitive ability. Although powerful for prediction, these techniques are not flexible, preventing the capture of more subtle relationships involving more than one category. The experimental approach uses less sophisticated analyses, primarily based on quartile splitting, to create a categorical variable from which the performance of extreme groups is compared (for detailed critiques of these practices, see, for example, Carroll, 1978; Farewell et al., 2004; Knüppel & Hermsen, 2010; Rouder & Haaf, 2019; Watkins, 2018). Both approaches have brought fruitful results, but no detailed inspection of the raw data is usually provided to support either the exclusion of data regarded as outliers or the relevance of the selected analyses. From this point of view, these practices seem questionable in that they prevent real exploration of individual differences, characterized by the noise they induce in the data. The k-means clustering algorithm, on the other hand, works without prior categories or information on the data. The only constraint is fixing the number of clusters before starting, and then the groups are created solely based on distance measurements between the data. Notwithstanding potential concerns about its accuracy and reliability when compared to the correlational approach, due to its sensitivity to initial conditions and the chosen number of clusters, k-means clustering remains a valuable technique for identifying distinct qualitative groups and revealing meaningful structures or patterns.

Unfortunately, and despite articles encouraging the use of algorithmic modeling as developed in machine learning (e.g., Yarkoni & Westfall, 2017) and descriptions of how to implement these models for research (e.g., Rosenbusch et al., 2021), behavioral studies of individual differences seem to be mostly limited to the use of traditional techniques such as factor analysis and parametric tests. This may be because research is more focused on prediction than on characterizing individual differences, but also because the results can be discussed with respect to a well-known, already existing, literature. Enhancing our understanding of the behavioral and cognitive processes that contribute to individual differences relies on acquiring more detailed descriptions of variation and embracing innovative methods of data analysis, such as data mining and machine learning. These techniques offer a comprehensive overview of the wide spectrum of individual differences and facilitate the identification of distinctive behaviors that form the essence of these variations. The objective of this application-oriented study is to encourage a more comprehensive exploration of behavioral responses and the utilization of clustering techniques to enhance the characterization of individual differences in cognitive abilities.

## Avenues for future research

The findings of this study are limited to the specific experimental design and sample size. Other factors, such as environmental, psychological, behavioral, and physiological characteristics, which are known to contribute to inter-individual variations in behavior, were not considered. The robustness of the obtained results would be enhanced by examination of the stability of the identified clusters under different test conditions. For example, changing the rate of target and lure stimuli, stimulus presentation time, responding time limit, or adding positive or negative monetary reinforcements or interferences by altering the shape-color association of the stimuli. However, conducting such an assessment requires the use of an across-subjects counterbalancing design to control for the order effect, which can be time-consuming and may induce fatigue, thereby introducing bias in the results. An alternative approach to achieve this goal is to use self-report measures to assess personality traits and approach and avoidance behaviors in a choice situation. Combining these measures with SDT responses would enable the identification of subgroups of participants exhibiting similar behavioral patterns, using a configurational frequency analysis (Von Eye, 2007). Such additional research would deepen our understanding of how individual variability can influence risk perception in decision-making situations.

### Supplementary information

Below is the link to the electronic supplementary material.

Supplementary file1 (PDF 220 KB)

## Data Availability

The raw data, SPSS syntax and R code used for this study are available at 10.5281/zenodo.7417342. This page provides all the elements used for this article.
