# Supplementary material for: Interindividual variations in associative visual learning: Exploration, description, and partition of response characteristics
Source: Behav Res Methods. 2023 Aug 24;56(5):4643–60. doi: 10.3758/s13428-023-02208-z (PMC11289025; doi:10.3758/s13428-023-02208-z)
Supplement: Supplementary file 1 — Supplementary file1 (PDF 220 KB) [file 13428_2023_2208_MOESM1_ESM.pdf]

SPSS Syntaxes

Raw data exploration

```
GLM M1 M2 M3 M4 M5
/WSFACTOR=Blocks 5 Difference
/METHOD=SSTYPE(3)
/EMMEANS=TABLES(OVERALL)

/EMMEANS=TABLES(Blocks) COMPARE ADJ(LSD)
/PRINT=DESCRIPTIVE ETASQ OPOWER
/CRITERIA=ALPHA(.05)
/WSDESIGN=Blocks.
```

Signal Detection Theory

Calculation of d' and *c* indexes

```
COMPUTE target = Hit+Miss.
COMPUTE lure = FA + CR.
COMPUTE usn=probit(Miss/target).
COMPUTE un=probit(CR/lure).
COMPUTE d'=UN-USN.
COMPUTE c=(UN+USN)/2.
EXECUTE .
```

Basic Statistical Descriptions of the Four SDT Responses

```
EXAMINE VARIABLES=Hit Miss CR FA
 /ID=ID
 /PLOT BOXPLOT STEMLEAF HISTOGRAM NPPLOT
 /COMPARE GROUPS
 /PERCENTILES(5,10,25,50,75,90,95)  HAVERAGE
 /STATISTICS DESCRIPTIVES EXTREME
 /CINTERVAL 95
 /MISSING LISTWISE
 /NOTOTAL.
```

Basic Statistical Descriptions of the Eight Response Characteristic

```
DATASET ACTIVATE DataSet1.
EXAMINE VARIABLES=Zcorrect_response_mean ZCV Zd' Zc ZSs ZSp ZTI ZTII
/PLOT BOXPLOT STEMLEAF NPPLOT
/COMPARE GROUPS
/STATISTICS DESCRIPTIVES
/CINTERVAL 95
/MISSING LISTWISE
/NOTOTAL.
```

Cluster Analysis, Centroid Model

K-Means Clustering Analysis of Eight Response Characteristics (Clustering A)

Agglomerative hierarchical clustering

```
DATASET ACTIVATE DataSet2.
CLUSTER   ZMean_CR ZCV_CR Zd' Zc ZSs ZSp ZTI ZTII
/METHOD WARD
/MEASURE=SEUCLID
/PRINT SCHEDULE
/PRINT DISTANCE
/PLOT DENDROGRAM VICICLE.
```

3K-means clustering

```
QUICK CLUSTER ZGM ZCV Zd' Zc ZSs ZTI ZSp ZTII
/MISSING=LISTWISE
/CRITERIA=CLUSTER(3) MXITER(10) CONVERGE(0)
/METHOD=KMEANS(NOUPDATE)
```

```
/SAVE CLUSTER DISTANCE
/PRINT ID(Subect) INITIAL ANOVA CLUSTER DISTAN.
```

Principal Component Analysis

```
FACTOR
/VARIABLES Mean CV d' c Ss Sp TI TII
/MISSING LISTWISE
/ANALYSIS Mean CV d' c Ss Sp TI TII
/PRINT UNIVARIATE INITIAL CORRELATION SIG KMO EXTRACTION ROTATION FSCORE
/FORMAT SORT
/PLOT EIGEN ROTATION
/CRITERIA FACTORS(2) ITERATE(25)
/EXTRACTION PC
/CRITERIA ITERATE(25)
/ROTATION VARIMAX
/SAVE REG(ALL)
/METHOD=CORRELATION.
```

K-Means Clustering of FS (Clustering B)

Agglomerative hierarchical clustering

```
DATASET ACTIVATE DataSet5.
CLUSTER   FAC1_1 FAC2_1
/METHOD WARD
/MEASURE=SEUCLID
/PRINT SCHEDULE
/PRINT DISTANCE
/PLOT DENDROGRAM VICICLE.
```

3K-means clustering

```
QUICK CLUSTER FAC1_1 FAC2_1
/MISSING=LISTWISE
/CRITERIA=CLUSTER(3) MXITER(10) CONVERGE(0)
/METHOD=KMEANS(NOUPDATE)
/SAVE CLUSTER DISTANCE
/PRINT ID(Subect) INITIAL ANOVA CLUSTER DISTAN.
```

ROC Space

K-Means Clustering of Two-Dimensional ROC Space (Clustering C)

Agglomerative hierarchical clustering

```
DATASET ACTIVATE DataSet6.
CLUSTER   ZSs ZTI
/METHOD WARD
/MEASURE=SEUCLID
/PRINT SCHEDULE
/PRINT DISTANCE
/PLOT DENDROGRAM VICICLE.
```

3K-means clustering

```
QUICK CLUSTER ZSs ZTI
/MISSING=LISTWISE
/CRITERIA=CLUSTER(3) MXITER(10) CONVERGE(0)
/METHOD=KMEANS(NOUPDATE)
/SAVE CLUSTER DISTANCE
/PRINT ID(ID) INITIAL ANOVA CLUSTER DISTAN.
```

Cross table clustering A X clustering B

```
DATASET ACTIVATE DataSet12.
CROSSTABS
/TABLES=Clustering_A BY Clustering_B
```

```
/FORMAT=AVALUE TABLES
/STATISTICS=CHISQ CC CORR
/CELLS=COUNT
/COUNT ROUND CELL.
```

Clustering Comparison

Cross table clustering A X clustering C

```
CROSSTABS

/TABLES=Clustering_A BY Clustering_C
/FORMAT=AVALUE TABLES
/STATISTICS=CHISQ CC CORR
/CELLS=COUNT
/COUNT ROUND CELL.
```

Cross table clustering B X clustering C

```
CROSSTABS
/TABLES=Clustering_B BY Clustering_C
/FORMAT=AVALUE TABLES
/STATISTICS=CHISQ CC CORR
/CELLS=COUNT
/COUNT ROUND CELL.
```

Tables

Clustering (A,B)	1	2	3	*N*
1	2	11	0	13
2	2	0	11	13
3	23	0	0	23
N	27	11	11	

Clustering (A,C)	1	2	3	*N*
1	12	0	1	13
2	0	4	9	13
3	0	0	23	23
N	12	4	33	

Clustering (A,C)	1	2	3	*N*
1	1	0	26	27
2	11	0	0	11
3	0	4	7	11
N	12	4	33	

R codes

Three typical indices used for comparison between partitions

# Comparing partitions (CB, 2022), R syntax

```
# reads the attributed groups resulting from three SPSS analyses A, B and C
data=read.csv2("ClustersSubjectsSPSS.csv")

# load the R package "partitionComparison"
library(partitionComparison)

# transforms the memberships into objects of class "Partition"
A = new("Partition", data$Clustering_A)
B = new("Partition", data$Clustering_B)
C = new("Partition", data$Clustering_C)

# creates cross-classification contingency tables
table(A,B)
table(A,C)
table(B,C)

# computes the Rand index, the Jaccard coefficient and Meila's index "Variation of Information"

randIndex(A,B)
jaccardCoefficient(A,B)
variationOfInformation(A,B)

randIndex(A,C)
jaccardCoefficient(A,C)
variationOfInformation(A,C)
randIndex(B,C)
jaccardCoefficient(B,C)
variationOfInformation(B,C)
```

k coefficient

```
# group 1
data1=dataALL[dataALL$Group==1,]

# effectifs cumules:
Hit1=sum(data1$Hit)
FA1=sum(data1$FA)
Miss1=sum(data1$Miss)
CR1=sum(data1$CR)

Nobserved1=as.table(matrix(data=c(Hit1,FA1,Miss1,CR1),2,2)); Nobserved1
Paccord1=sum(diag(Nobserved1))/sum(Nobserved1); Paccord1 # 0.6122685
Prandom1=sum(diag(chisq.test(Nobserved1)$expected))/sum(chisq.test(Nobserved1)$expected); Prandom1
#  0.5

kappa1=(Paccord1-Prandom1)/(1-Prandom1); kappa1 # 0.224537 : Cohen Kappa for group 1

# groupe 2
data2=dataALL[dataALL$Group==2,]

# effectifs cumules:
Hit2=sum(data2$Hit)
FA2=sum(data2$FA)
Miss2=sum(data2$Miss)
CR2=sum(data2$CR)
```

R codes

```
Nobserved2=as.table(matrix(data=c(Hit2,FA2,Miss2,CR2),2,2)); Nobserved2
Paccord2=sum(diag(Nobserved2))/sum(Nobserved2); Paccord2 # 0.6243056
Prandom2=sum(diag(chisq.test(Nobserved2)$expected))/sum(chisq.test(Nobserved2)$expected); Prandom2
# 0.5

kappa2=(Paccord2-Prandom2)/(1-Prandom2); kappa2 # 0.2486111 : Cohen Kappa for group 2

# groupe 3
data3=dataALL[dataALL$Group==3,]

# effectifs cumules:
Hit3=sum(data3$Hit)
FA3=sum(data3$FA)
Miss3=sum(data3$Miss)
CR3=sum(data3$CR)

Nobserved3=as.table(matrix(data=c(Hit3,FA3,Miss3,CR3),2,2)); Nobserved3
Paccord3=sum(diag(Nobserved3))/sum(Nobserved3); Paccord3 # 0.7996633
Prandom3=sum(diag(chisq.test(Nobserved3)$expected))/sum(chisq.test(Nobserved3)$expected); Prandom3
# 0.5

kappa3=(Paccord3-Prandom3)/(1-Prandom3); kappa3 # 0.5993266: Cohen Kappa for group 3
```
